# Lysine and Methionine Supplementation for Dairy Calves Is More Accurate through the Liquid than the Solid Diet

**DOI:** 10.3390/ani11020332

**Published:** 2021-01-28

**Authors:** Jackeline Thais Silva, Evangelina Miqueo, Thaís Manzoni Torrezan, Nathalia Brito Rocha, Giovana Simão Slanzon, Gercino Ferreira Virginio Júnior, Carla Maris Machado Bittar

**Affiliations:** Luiz de Queiroz College of Agriculture, Department of Animal Science, University of São Paulo, Av. Pádua Dias, 11. Piracicaba, São Paulo 13418-900, Brazil; jtsilva@ymail.com (J.T.S.); evangelina.miqueo@gmail.com (E.M.); manzoni.thais@gmail.com (T.M.T.); n.britorocha@yahoo.com.br (N.B.R.); giovana.slanzon@gmail.com (G.S.S.); gercino.ferreiravj@yahoo.com.br (G.F.V.J.)

**Keywords:** amino acids, blood metabolites, calf, glucose, pre-weaning

## Abstract

**Simple Summary:**

Amino acids (AA) are known to be a factor limiting animals’ growth when requirements are not achieved, specially lysine (Lys) and methionine (Met). However, requirements of these AA are not well established for dairy calves. This study aimed to evaluate the performance and metabolic changes in dairy calves supplemented with Lys and Met in milk replacer (MR) or starter concentrate (SC) according to doses recommended in the literature. The supplementation with Lys and Met did not benefit dairy calves’ performance nor metabolism. However, supplementation through the MR was more efficient than SC to result in adequate daily intakes of AA. Further studies are needed to understand the negative effects of AA on calf starter intake.

**Abstract:**

This study aimed to evaluate the performance and metabolic changes in dairy calves supplemented with lysine and methionine in milk replacer (MR) or starter concentrate (SC). Male Holstein calves (*n* = 45) were blocked and distributed in Control without supplementation (1) and; Lysine and Methionine supplementation to achieve an intake of 17 and 5.3 g/d in the SC (2) and to achieve of 17 and 5.3 g/d in the MR (3). MR was fed (6 L/d) until the 8th week of life when weaning occurred. Calves were followed until the 10th week of age. Feed intake was measured daily. Weight and body measurements were registered weekly. Blood samples were collected biweekly to evaluate the intermediate metabolism. The AA supplementation resulted in lower body weight at weaning and week 10. Calves fed SC Lys:Met had lower SC intake and lower total feed intake at weaning when compared to control. Calves fed control had higher heart girth, hip-width, and plasma glucose concentration. The supplementation with Lys and Met did not benefit dairy calves’ performance nor metabolism in this study. Supplementation through the MR was more efficient than SC to result in adequate daily intakes of AA. Further studies are needed to understand the negative effects of AA on calf starter intake.

## 1. Introduction

Recent research has shown the benefits of increasing weight gain of dairy calves, mainly by feeding higher levels of liquid diet [1,2], even if there is an impact on the raising daily costs. Feeding with milk replacer (MR) can be an alternative to reduce costs, but it can result in different performance when feeding the same volumes due to differences in crude protein (CP) and fat content when compared to whole milk [3]. Furthermore, the inclusion of non-dairy protein sources can cause amino acid imbalance and difficulty in meeting the calves’ needs to achieve a desirable growth rate [4,5,6].

The amino acid requirements for calves are not well defined. The industry uses values estimated in a limited number of studies, with calves fed whole milk and no access to starter concentrate [7,8,9]. According to Gerrits [10], the rate of protein deposition may be limited by one or more AA. Lysine (Lys) and methionine (Met) are always cited as the main limiting amino acid (AA) for the potential growth of milk replacer-fed calves rather than whole milk [7,11,12,13]. However, threonine and isoleucine can also be considered, especially when non-dairy protein sources are used in MR formulation [6,14].

Nevertheless, the recommendation of 17 g/d Lys and 5.3 g/d Met for calves under 5 weeks of age has proved to be adequate to meet the requirements of calves in the study by Hill et al. [12], supplementing MR containing 24% to 28% CP and 17% fat. Depending on the supplemented AA, there may be an increase in the average daily gain (ADG) [12,15] and positive or no response on growth [4,16,17].

Even when calves are fed whole milk, considered the best source of protein due to its high digestibility and AA profile, the Met concentration is below ideal levels for improved calf performance [4,12,15]. Besides, calves fed a medium CP milk replacer do not meet the requirements recommended by Hill et al. [12] when moderate volumes such as 6 L/d are fed [3]. Other studies suggested that the industry should consider synthetic AA supplementation in MR formulation to improve the performance of dairy calves [3,15].

Solid feed intake can also provide AA for intestinal absorption. However, starter concentrate (SC) intake is low for young calves and is negatively correlated with the MR feeding level [18]. As SC intake increases, ruminal development begins, and calves can use microbial protein (MP) to meet AA requirements. Several changes in protein metabolism occur as a result of ruminal development [19]. These changes affect the bacterial population and consequently affect the composition of the MP and the percentage of N reaching the abomasum and the intestine. According to Quigley et al. [19], bacterial N represents about 45% of the total N reaching the abomasum, when calves present a starter intake of around 1 kg/d. However, even for adult dairy animals, the production of the MP that reaches the small intestine can be low and supplementation of Lys and Met improves performance [20].

Most of the studies have evaluated the AA supplementation in MR. However, AA can be included in starter in an attempt to improve MP synthesis [21,22,23], and consequently the MP that reaches the intestine can be used by the animals [24]. In addition, supplementation of hydroxyl-analogue AA may increase absorption through the rumen wall [25]. However, solid feed intake has a high coefficient of variation [26], resulting in difficulties to achieve adequate AA intake.

This study aimed to evaluate the effect of adding Lys and Met in the milk replacer or the starter concentrate, on AA daily intake, performance and metabolism of dairy calves.

## 2. Materials and Methods

All experimental procedures were approved by the Animal Care and Use Committee of the University of São Paulo and followed the guidelines recommended (Protocol numeber 2013-8).

This study was conducted at the calf facilities of the Department of Animal Science, “Luiz de Queiroz” College of Agriculture, University of Sao Paulo, located in Piracicaba–Sao Paulo, Brazil. The trial period was from January to June 2013. During this period, the average temperature was 23.1 °C, with a maximum of 28.9 °C and a minimum of 17.2 °C. The mean relative humidity during the study period was 82%, and the average rainfall was 149.3 mm/mo.

Forty-five male Holstein calves from a commercial farm (2 ± 2 d of age), were used. Calves were immediately separated from their dam at birth, weighed, and fed 2 L of unpasteurized high-quality colostrum (>50 g/L of IgG) in the first two meals by bottle, resulting in an intake of 4 L within 10 h of age. When fresh colostrum was not adequate in quality or volume, frozen non-pooled stored colostrum was thawed for the newborn. Only animals with serum protein above 5.5 g/dL at 48 h of life were enrolled in the study, as suggested by the literature as a cutoff for adequate passive transfer [27]. Calves were transported to the experimental calf facility at the “Luiz de Queiroz” College of Agriculture, University of São Paulo (approximately 100 km), and housed in individual wood shelters (1.35 m height, 1 m width and 1.45 m depth) distributed in a trimmed grassy field. Calves were contained with a chain belt attached to a thin chain, allowing an adequate walking area, but no physical contact with each other.

The animals had free access to water and a commercial finely ground SC during the entire experimental period. Calves were bucket-fed with 6 L/d (730 g DM/d) of a commercial milk replacer (21.6 CP:13.8 fat, diluted to 12.5% solids; Violet Sprayfo^®^, Sloten do Brasil Ltd.a., Santos, SP, Brasil; São Paulo, Brazil), divided into two meals (07 h and 17 h), from the 2nd day of life until the eighth week of life, when calves were abruptly weaned. Abrupt weaning was applied and hay was not fed after that to do not confound AA intake during and after the weaning process. The medium CP milk replacer was chosen because it was the most adopted by producers at the time of the study, suggesting an opportunity for AA supplementation for this type of commercial product. The feed intake was measured daily by weighing the feed offered and orts.

Calves were enrolled in the study from the second half of January to the first half of March, as cows calved. Calves were blocked considering the birth date and weight and randomly assigned to treatments. Treatments differ in Lys and Met supplementation as follows: (1) Control: MR and SC without AA supplementation (*n =* 15); (2) SC Lys:Met: ground SC with the addition of Lys and Met to achieve a consumption of 17 and 5.3 g/d, respectively, with correction based on the analysis of the SC composition (*n =* 15); (3) MR Lys:Met: MR with the addition of Lys and Met to achieve a consumption of 17 and 5.3 g/d, respectively, with correction based on the analysis of the MR composition (*n =* 15).

For the determination of amino acid profile in feeds, a near infrared spectroscopy (NIRS) calibration curve for dairy products validated by Adisseo Brazil Animal Nutrition Ltd.a. was used. Based on this analysis, SC and MR were supplemented until reaching the recommended levels of Lys and Met by Hill et al. [12]. For calculation, a total intake of 6 L/d of the MR and an average of 800 g of SC intake during the whole period were considered. Milk replacer evaluation showed a concentration of 1.09 g/100 g DM of Lys and 0.306 g/100 g DM of Met, resulting in a total intake of 7.9 g of Lys and 2.23 g of Met. To correct the gap for the total intake of Lys (17 − 7.9 = 9.04 g) and of Met (5.3−2.23 = 3.07 g), the MR was supplemented by adding 309 g of L-lysine (Ajinomoto do Brazil Ind. e Com. de Alimentos Ltd.a, São Paulo, Brazil) and 105 g de DL-methionine (110 g de Rhodomet, Adisseo for each 25 kg of MR. Thus, MR was corrected to have 12.6 g/kg and 4.2 g/kg of Lys and Met, respectively, allowing the total planned intake to be reached by feeding 730 g of MR. The same rationale was applied for the SC. Starter concentrate analysis resulted in a concentration of 1.40 g/100 g DM of Lys and 0.50 g/100 g DM of Met, leading to a total intake of 11.2 g/d of Lys and 4.0 g/d of Met, considering an average intake of 800 g/d of concentrate. To supplement the difference for the total intake of Lys (17−11.2 = 5.8 g) and of Met (5.3−4.0 = 1.3 g), the starter was supplemented by adding 171.6 g L-lysine (AjiLys^®^ 99, Ajinomoto) and 40.59 g DL-methionine (Rhodimet, Adisseo, São Paulo, SP, Brazil) for each 25 kg of starter. Therefore, SC was corrected to have 6.86 g/kg and 1.62 g/kg of Lys and Met, respectively, allowing the total planned intake to be reached with an average SC intake of 800 g/d.

Feed samples were collected during the experimental period for further analysis (Table 1). The samples of feed were oven dried (model MA035-Marconi, Piracicaba, Brazil) at 55 °C for 72 h and ground through a 1 mm in Wiley mill (Marconi, Piracicaba, Brazil). The dry matter (DM) was determined by drying the samples in an oven at 105 °C for 24 h and mineral matter (MM) by incinerating the samples in muffle at 550 °C for 4 h (AOAC, 2012). The total nitrogen concentration was determined using the Leco TruMac^®^ N apparatus (Leco Corporation, St. Joseph, MI, USA; AOAC, 2012). The crude protein (CP) was calculated by multiplying the total nitrogen by 6.25. The extract ether concentration was determined according to AOAC [28]. The gross energy was determined by calorimetry (PARR 1261, Parr Instrument Company, Moline, IL, USA).

The animals were weighed weekly on a mechanical scale before the morning feeding, and growth measures were taken using a ruler for hip-width and withers height, and a flexible tape for heart girth until the 10th week of life when the study ended. The fecal score was monitored daily, as described by Larson et al. [29], regarding the fluidity of feces: (1) normal and firm, (2) loose but with general healthy aspect, (3) very loose not watery separation, and (4) watery. When a fecal score was higher than 2, calves were rehydrated with an oral electrolyte solution. Health problems were monitored and treated according to veterinary recommendations.

Blood samples were collected once at weeks 2, 4, 6, 8, and 10, always 2 h after the morning feeding, through jugular venipuncture, into vacuum tubes containing sodium fluoride and potassium EDTA (Vacuette, Campinas, Brazil) and vacuum tubes containing clot activator and gel (Vacuette, Campinas, Brazil). The samples were centrifuged (Universal 320R, Hettich, Tuttlingen, Germany) at 2000× *g*, for 20 min at 4 °C. Plasma and serum were freezers stored (−26 °C) until further analysis. Specific commercial enzymatic kits (Labtest Diagnóstica S.A., Lagoa Santa, Brazil) were used to analyze plasma glucose, total serum protein (TSP), serum albumin, alkaline phosphatase, and serum creatinine. β-hydroxybutyrate (BHBA) was determined using a commercial kit RANBUT (Randox Laboratories, Life Sciences Ltd.a., Crumlin, UK). Plasma urea nitrogen (PUN) determination was performed according to the colorimetric method [30], adapted for reading in Automatic System for Biochemistry-Model SBA 200 (CELM, Barueri, Brazil).

All data were tested for normal distribution by the Shapiro-Wilk test, and homogeneity of the variances using the Levene test. The data were analyzed using the MIXED procedure of the SAS statistical package (version 9.4, SAS Institute Inc., Cary, NC, USA), with the collection days as repeated measures (model 1). The best covariance structure was identified from different covariance structures by comparing the AICC statistic (Akaike Information Criteria Corrected). The differences were considered significant at *p* < 0.05. Treatments comparison was carried out according to the adjusted Tukey test.

Model (1): $Y_{ijk}=\mu +T_{i}+I_{j}+TI_{ij}+B_{k}+e_{ijk}$

Where: *Y_ijk_* = dependent variable; *μ* = overall average; *T_i_* = Treatment Effect; *I_j_* = effect of age; *TI_ij_* = interaction Treatment × Age; *B_k_* = Block effect; *e_ijk_* = experimental error.

## 3. Results

The average starter concentrate intake throughout the pre-weaning phase increased with the age of the calves (*p* < 0.05), but was not affected by supplementation or by the interaction between age and supplementation (Table 2). The starter concentrate intake at weaning (8th week) was higher for the animals without supplementation (control) as compared to those supplemented in the SC, without differing from those supplemented in the MR (*p* < 0.05). At the 10th week, the starter concentrate intake was similar among treatments. However, overall starter intake was below the expected 800 g/d regardless of treatments (336.5 g/d).

**Figure 1:**
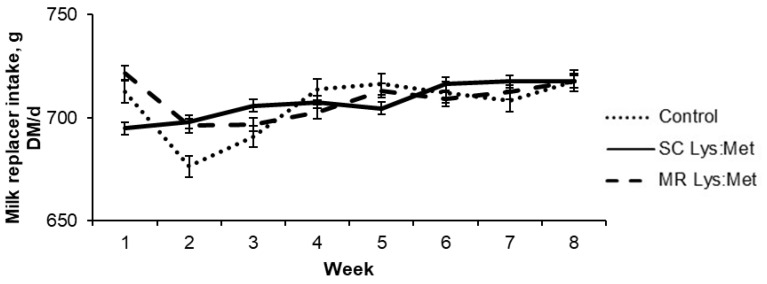
Milk replacer intake in dairy calves with or without lysine and methionine supplementation in starter concentrate or milk replacer. Error bars indicate standard error of the mean.

Milk replacer intake was variable in the first weeks of life, although the supply was constant (730 g DM/d; Figure 1). There was a reduction between the first and second to third weeks of life (Figure 1). After the second week of age, no refusals were observed for any of the treatments.

The total feed intake (SC + MR) in the pre-weaning phase also increased with age (*p* < 0.05), but was not affected by the supplementation or the interaction between age and supplementation (Table 2). However, after weaning, the control animals had a higher total feed intake, as compared to the animals fed with the SC Lys:Met, without differing from the animals fed with the MR Lys:Met (*p* = 0.05).

**Figure 2:**
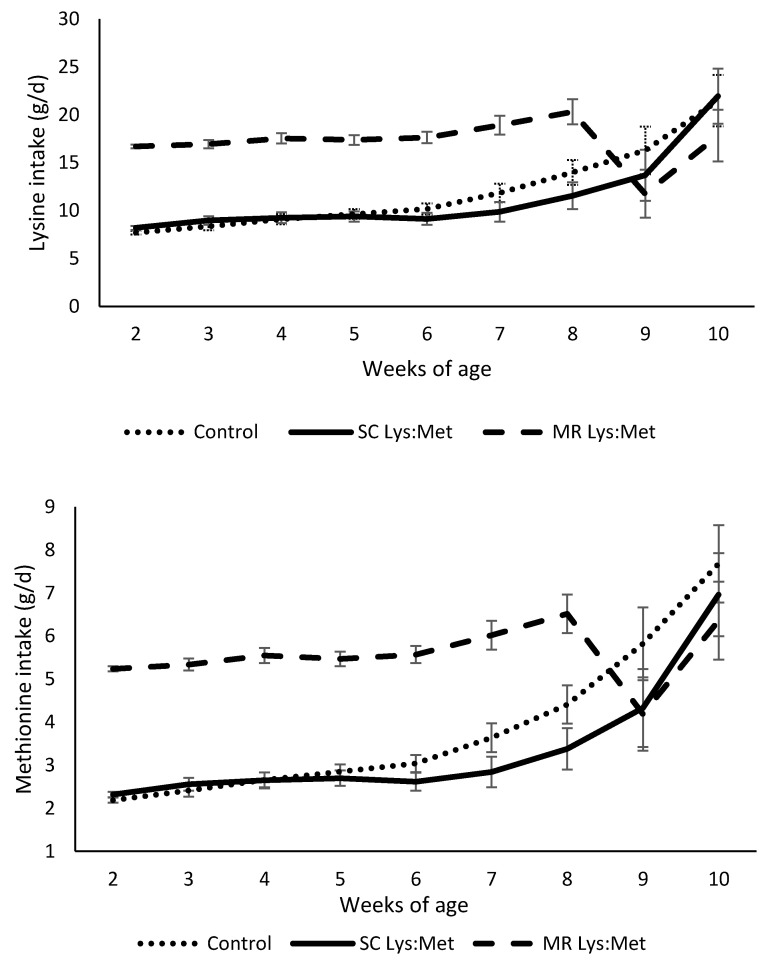
Lysine and methionine intake of dairy calves with or without lysine and methionine supplementation in starter concentrate or milk replacer. Calves fed milk replacer supplemented with Lys and Met, presented a higher intake of both amino acids during the pre-weaning period (week 2 to 8) with *p <* 0.01. Error bars indicate standard error of the mean.

Differences in SC intake and fixed MR feeding affected total Lys and Met intake during the pre-weaning period (Table 2 and Figure 2; *p* < 0.01). Feeding MR Lys:Met resulted in higher Lys and Met intake, aiding calves to meet recommendations for these AA.

**Figure 3:**
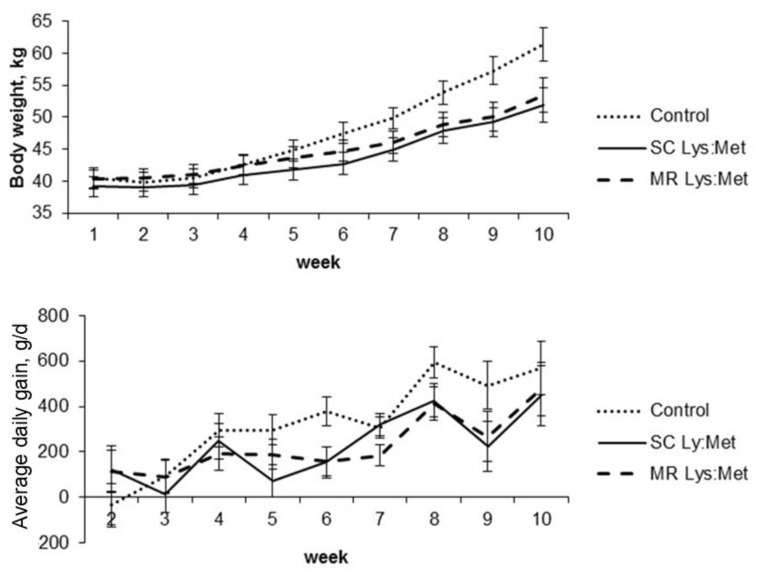
Body weight (kg) and average daily gain (g/d) of dairy calves with or without lysine and methionine supplementation in starter concentrate or milk replacer. Error bars indicate standard error of the mean.

Body weight was higher for the animals fed the control compared to the animals in SC Lys:Met diet, without differing from those fed with MR Lys:Met in the whole period (*p* < 0.05). Body weight increased with age (*p* < 0.05). There was also a significant effect of the interaction between treatment and age (*p* < 0.05; Table 3), with animals in the control group with greater body weight after the sixth week of age, while supplemented calves did not differ in this parameter throughout the experimental period (*p* < 0.05; Figure 3). Besides, animals fed with SC Lys: Met had lower average body weight (from birth to the 10th week) compared to animals fed with control (*p* < 0.05), with no differences between animals fed with MR Lys: Met. The body weight at weaning and the 10th week were also higher for animals in the control group compared to supplemented animals (*p* < 0.05).

The supplementation did not affect pre or post-weaning average daily gain with values of 195.6 and 370.9 g/d, respectively. Although the effect of age was significant (*p* < 0.05), there was no interaction between supplementation and age (Table 3). Amino acid supplementation *per se* did not affect withers height, however there was a treatment by age interaction (Table 3). However, hip-width was greater for the animals in the control group compared to supplemented animals (*p* < 0.05). The animals fed SC Lys:Met showed a smaller heart girth than the control group, but similar to that of MR Lys:Met. All measures increased with age (*p* < 0.05). Only the withers height was affected by the interaction between supplementation and age, with higher values for control calves only after weaning (*p* < 0.05).

**Figure 4:**
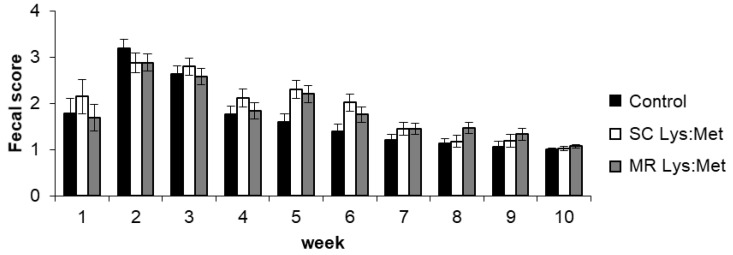
Fecal score of dairy calves with or without lysine and methionine supplementation in starter concentrate or milk replacer. Error bars indicate standard error of the mean.

In this study, it was considered that the animal was affected by diarrhea when the fecal score was >2.5. The highest incidence of diarrhea occurred during weeks 2 and 3 in all treatments (Figure 4). While control calves had fecal scores >2 only at weeks 2 and 3, SC Lys:Met calves still had high fecal scores until the 6th week of age. However, no treatment, age, or interaction of these factors were significant (*p* > 0.05).

**Figure 5:**
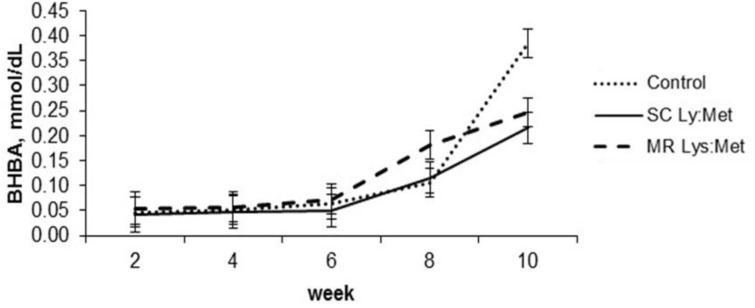
β-Hydroxybutyrate (BHBA) concentration in dairy calves with or without lysine and methionine supplementation in starter concentrate or milk replacer. Error bars indicate standard error of the mean.

The diets affected only the plasma glucose concentration, with higher concentration for the animals fed in the control group as compared to the animals fed with the MR Lys:Met, without differing from the SC Lys:Met (*p* < 0.05; Table 4). All blood metabolites were affected by age (*p* < 0.05). However, only BHBA was affected by the interaction of supplementation with age (Table 4 and Figure 5; *p* < 0.05). At the 10th week, control-fed calves had higher BHBA concentrations

## 4. Discussion

The SC intake was lower than expected, which negatively affected the intake of AA, since the average intake of 800 g/d considered for AA supplementation in the starter was not achieved. According to Quigley [31], calves can consume an average of 680 to 700 g/d of SC at weaning. Since calves were being followed until the 10th, an average of 800 g/d was considered. The literature shows that increased volumes of liquid diet may decrease the starter intake; however, this effect is clear for intakes higher than 750–800 g DM/d [18]. In the present study, calves were fed 730 g DM/d of milk replacer, which should not depress starter intake. Other studies from our laboratory have shown an average around 800 g/d of starter intake during a 10 wk period of evaluation [32,33,34].

Intakes of Lys and Met were affected pre-weaning and consequently during the whole period. Planned AA intakes were observed only for calves that were supplemented through MR, because of the fixed volumes fed and very little refusals (only during the first two weeks). The study approached two strategies to increase AA intake, through milk replacer, disregarding the AA content in the starter intake, and vice versa. Considering just part of the diet to correct AA intake may result in errors since calves will consume AA from both MR and SC. However, as a practical prospective, it would be easier to supplement only the MR, since the intake is fixed (or more predictable) as compared to SC. Furthermore, supplementation via MR would make AA available in the intestines because of the esophageal groove closure. On the other hand, when AA is supplemented through the starter concentrate, part of it may be available and used in the rumen (not in younger ages because rumen is not fully working) and part will escape and reach the intestine [19]. We expected a different effect for calves supplemented via concentrate as animals aged, because of rumen development and a shift in the use of the supplemented AA (less in the intestine and more in the rumen). Supplementation of AA should target the intestines when aiming the achievement of the ruminant requirements. However, AA supplementation through SC have depressed its intake. The data of AA intake shows that supplementation through the milk replacer is more feasible since intake is more predictable and controlled, and that calves were able to achieve the intake levels suggested by Hill et al. [12].

Excess, deficiency, or imbalance of amino acids decreases consumption in non-ruminant animals [35]. However, in a series of studies by Hill et al. [12], AA supplementation had no effect on starter concentrate intake when Met and Lys were included in a proportion of 3.25 (study 1), but it had a quadratic effect when Met inclusion was increased (0.64%, 0.68% and 0.72%, study 2). Silva et al. [17] also found no effect on SC intake when Lys and Met were supplemented in combination with glutamate and glutamine for calves pre and post-weaning. In the present study, after weaning, the SC intake increased and quickly reached the expected values; however, the average intake was still lower for calves supplemented through SC, suggesting a different effect than that related to supplementation level. Post-weaning proportion intakes were 2.79, 3.15, and 2.8 for calves on control, SC Lys:Met and MR Lys:Met, respectively. Feed intake was more affected postweaning, but Lys:Met ratio may not be the reason for that because it is within recommended levels [20]. Other factors such as solid feed particle size may play a role in depressing feed intake [36]. Since the starter was grounded, we may postulate that, together with AA supplementation trough SC, the proportion of fines may have affected intake. Unfortunately, we have not measured the particle size distribution of the SC.

In the first weeks of life, there was a small variation in MR intake for calves in the MR Lys:Met treatment, perhaps associated with an increased incidence of diarrhea, as suggested by the highest fecal scores. However, fecal scores were not different among treatments. Diarrhea is the main factor that influences the performance of the calf during the first weeks of life. As it is associated with high mortality rates, diarrhea is also responsible for decreasing the intake of liquid diet and SC, leading to weight loss or decreased ADG [37,38]. However, no differences among treatments were observed for fecal scores, nor the interaction between treatment and age.

Because of the depressed SC intake, ADG of calves supplemented with Lys and Met was lower than expected for pre-weaning calves fed 6 L/d of MR [12,16,39]. The CP level of the MR may have also limited ADG, even for calves supplemented with AA. Feeding medium CP milk replacers is common in some regions where higher CP products are not available. Bittar et al. [3] have shown that in Brazil the commercially available products averaged 21.2% of CP, with the highest product containing 24.9% CP. Furthermore, those data showed that fat % is also lower, but achieved more easily the NRC [9] recommendations. The authors [3] have concluded that calves fed 6 L/d, would not meet the Lys and Met requirements proposed by Hill et al. [12], suggesting that synthetic AA supplementation could be done. However, in the present study AA supplementation had no effect on ADG, suggesting that AA supplemented forms and utilization by calves need to be further studied.

The low SC intake may have also limited ruminal development, as evidenced by the BHBA concentration, decreasing the opportunity to show effects of AA supplementation through SC. However, all blood metabolites were affected by age, mainly due to changes in metabolism because of rumen development, as a response to increased SC intake. As plasma glucose decreased, BHBA concentrations increased, especially after weaning, suggesting a transition period from pre to the functional ruminant. Unlike the concentration of glucose, the concentration of BHBA should increase with age, in response to increased SC intake, which serves as another indicator of ruminal development [40]. In the 10th week, the calves fed with control had higher concentrations of BHBA, corroborated by the higher SC intake.

Calves were weaned regardless of SC intake, which is not recommended for successful weaning, even though others have shown that calves easily adapt to new feed management [33]. As the SC intake was low at weaning, but rapidly increased thereafter, the increases in BHBA concentrations were more pronounced at this point. This effect can be observed mainly among calves fed with SC Lys:Met, which was weaned with an SC intake of 197.7 g/d, and rapidly increased the SC intake and BHBA concentration. As shown in the present study, other studies with dairy calves reported an increase in the concentration of BHBA before weaning, with a marked increase after weaning [41,42]. Previous work by our laboratory evaluating supplementation with Lys and Met, in combination with glutamate and glutamine, for calves fed with MR, showed similar results [17].

## 5. Conclusions

Supplementation with lysine and methionine in the milk replacer (21.6% CP and 13.8% fat) or starter concentrate (20.5% CP) did not benefit the performance or metabolism of dairy calves, mainly because it reduced solid feed intake. However, supplementation via milk replacer was more effective in increase AA intake, since intake is less variable and more predictable. The changes observed in selected blood parameters were a result of increases in starter intake and the consequent rumen development as animals aged. Further studies are needed to understand the negative effects of synthetic AA on calf starter intake.

## Figures and Tables

**Table 1:** Chemical composition of milk replacer and starter, with or without lysine and methionine supplementation.

Parameter	Milk Replacer	Milk Replacer Lys:Met	Starter	Starter Lys:Met
Dry matter (%)	95.72	93.96	91.13	90.73
Ash (%DM)	8.14	9.68	6.56	6.40
Crude protein (%DM)	21.57	22.32	20.50	21.40
Crude fat (%DM)	13.79	14.84	6.11	5.92
Gross energy (cal/kg)	4360.12	4409.04	4072.03	4059.85

Notes: Milk replacer: Milk replacer without lysine and methionine supplementation; Milk replacer Lys:Met: Milk replacer supplemented with lysine and methionine; Starter: Starter concentrate without lysine and methionine supplementation; Starter Lys:Met: Starter concentrate supplemented with lysine and methionine. Starter ingredients composition as % of DM: 32.30% ground corn, 29.2% soybean meal, 15% wheat meal, 8.5% soybean expeller, 10% pregelatinized corn, 5% mineral and vitamin supplement.

**Table 2:** Intake of starter, total feed, lysine and methionine of dairy calves with or without lysine and methionine supplementation in starter or in milk replacer.

Parameter	Treatment			SEM	*p* Value		
	Control	SC Lys:Met	MR Lys:Met		T	A	TxA
**Starter concentrate intake (g/d)**							
Whole period	436.7	260.7	312.1	65.41	0.13	<0.05	0.24
At weaning (8th week)	433.4 ^a^	197.7 ^b^	243.3 ^ab^	55.13	<0.05	--	--
Tenth week	1542.6	1059.4	1265.3	161.34	0.10	--	--
Total feed intake * (g DM/d)							
Pre-weaning	881.6	800.1	807.3	40.44	0.27	<0.05	0.18
Post-weaning	1354.2 ^a^	856.4 ^b^	1048.8 ^ab^	151.59	0.05	<0.05	0.74
**Pre-weaning AA intake, (g/d)**							
Lysine	10.14 ^b^	9.58 ^b^	17.84 ^a^	0.657	<0.01	<0.01	0.69
Methionine	3.03 ^b^	2.75 ^b^	5.64 ^a^	0.220	<0.01	<0.01	0.56
**Post-weaning AA intake, (g/d)**							
Lysine	18.85	17.81	14.81	11.702	0.43	<0.01	0.42
Methionine	6.74	5.64	5.27	0.855	0.38	<0.01	0.64
**Whole period AA intake, (g/d)**							
Lysine	11.94 ^b^	10.96 ^b^	17.45 ^a^	1.257	0.01	<0.01	<0.01
Methionine	3.81 ^b^	3.24 ^b^	5.66 ^a^	0.427	0.01	<0.01	<0.01

SEM, Standard error of mean; T, treatment effect; A, age effect (week); T×A, interaction treatment × age; Control: basal diet without supplementation with amino acids; SC Lys: Met: starter concentrate supplemented with lysine and methionine; MR Lys: Met: milk replacer supplemented with lysine and methionine; ^a,b^ Means values with different superscripts within the same parameter are different at *p* < 0.05; * Solid and liquid diet.

**Table 3:** Body weight, average daily gain and corporal measures of dairy calves with or without lysine and methionine supplementation in starter or milk replacer.

Parameter	Treatment			SEM	*p* Value		
	Control	SC Lys:Met	MR Lys:Met		T	A	TxA
**Body weight (kg)**							
Initial	40.5	39.1	40.3	1.51	0.84	--	--
At weaning	53.8 ^a^	47.9 ^b^	48.8 ^b^	1.90	<0.05	--	--
10th week	61.4 ^a^	51.8 ^b^	53.4 ^b^	2.64	<0.05	--	--
Whole period	47.8 ^a^	43.7 ^b^	45.1 ^a,b^	1.64	<0.05	<0.05	<0.05
**Average daily gain (g)**							
Pre-weaning	245.2	168.6	173.2	31.47	--	--	--
Post-weaning	539.7	214.2	358.8	10.56	--	--	--
Whole period	306.9	180.6	212.2	38.42	0.06	<0.05	0.19
**Average corporal measures (cm)**							
Withers height	83.5	81.6	82.6	0.57	0.10	<0.05	<0.05
Heart girth	83.4 ^a^	81.0 ^b^	82.2 ^a,b^	0.62	<0.05	<0.05	0.17
Hip width	23.4 ^a^	22.4 ^b^	22.8 ^b^	0.21	<0.05	<0.05	0.31

SEM, Standard error of mean; T, treatment effect; A, age effect (week); T×A, interaction treatment × age; Control, basal diet without supplementation with amino acids; SC Lys:Met, starter concentrate supplemented with lysine and methionine; MR Lys:Met, milk replacer supplemented with lysine and methionine; ^a,b^ Means values with different superscripts within the same parameter are different at *p* < 0.05.

**Table 4:** Blood metabolites of dairy calves with or without lysine and methionine supplementation in starter or milk replacer.

Blood Metabolite	Treatment			SEM	*p* Value		
	Control	SC Lys:Met	MR Lys:Met		T	A	TxA
Glucose (mg/dL)	91.9 ^a^	89.8 ^ab^	84.2 ^b^	2.33	0.05	<0.05	0.40
BHBA (mmol/L)	0.13	0.09	0.12	0.01	0.18	<0.05	<0.05
Alkaline phosphatase (U/L)	135.5	114.6	111.0	10.67	0.16	<0.05	0.15
Creatinine (mg/dL)	0.83	0.81	0.84	0.03	0.73	<0.05	0.13
Total protein (g/dL)	5.4	5.4	5.3	0.12	0.74	<0.05	0.88
Albumin (g/dL)	2.5	2.4	2.5	0.04	0.07	<0.05	0.53
N-ureic (mg/dL)	11.3	11.1	11.9	0.48	0.49	<0.05	0.51

SEM, Standard error of mean; T, treatment effect; A, age effect (week); T×A, interaction treatment × age; Control, basal diet without supplementation with amino acids; SC Lys:Met, starter concentrate supplemented with lysine and methionine; MR Lys:Met, milk replacer supplemented with lysine and methionine; ^a,b^ Means values with different superscripts within the same parameter are different at *p* < 0.05.

## Data Availability

The data presented in this study are available on request from the corresponding author. The data are not publicly available due to restrictions by the research group.

## References

[1] Soberon F., Van Amburgh M.E. (2013). Lactation Biology Symposium: The effect of nutrient intake from milk or milk replacer of preweaned dairy calves on lactation milk yield as adults: A meta-analysis of current data1. J. Anim. Sci..

[2] Alimirzaei M., Alijoo Y.A., Dehghan-Banadaky M., Eslamizad M. (2020). The effects of feeding high or low milk levels in early life on growth performance, fecal microbial count and metabolic and inflammatory status of Holstein female calves. Animal.

[3] Bittar C.M.M., da Silva J.T., Chester-Jones H. (2018). Macronutrient and amino acids composition of milk replacers for dairy calves. Rev. Bras. Saúde e Produção Anim..

[4] Castro J.J., Hwang G.H., Saito A., Vermeire D.A., Drackley J.K. (2016). Assessment of the effect of methionine supplementation and inclusion of hydrolyzed wheat protein in milk protein-based milk replacers on the performance of intensively fed Holstein calves. J. Dairy Sci..

[5] Morrison S.Y., Campbell J.M., Drackley J.K. (2017). Amino acid supplementation of calf milk replacers containing plasma protein. J. Dairy Sci..

[6] Vasquez K.M., Morrison S.Y., Campbell J.M., Drackley J.K. (2017). Plasma protein and supplemental isoleucine in milk replacers for dairy calves. J. Dairy Sci..

[7] Williams A.P., Hewitt D. (1979). The amino acid requirements of the preruminant calf. Br. J. Nutr..

[8] Gerrits W.J.J., France J., Dijkstra J., Bosch M.W., Tolman G.H., Tamminga S. (1997). Evaluation of a Model Integrating Protein and Energy Metabolism in Preruminant Calves. J. Nutr..

[9] NRC (2001). Nutrient Requirements of Dairy Cattle.

[10] Gerrits W.J.J. (2019). Symposium review: Macronutrient metabolism in the growing calf. J. Dairy Sci..

[11] Tzeng D., Davis C.L. (1980). Amino Acid Nutrition of the Young Calf. Estimation of Methionine and Lysine Requirements. J. Dairy Sci..

[12] Hill T.M., Bateman H.G., Aldrich J.M., Schlotterbeck R.L., Tanan K.G. (2008). Optimal Concentrations of Lysine, Methionine, and Threonine in Milk Replacers for Calves Less than Five Weeks of Age. J. Dairy Sci..

[13] Li H., Diao Q.Y., Zhang N.F., Fan Z.Y. (2008). Growth, Nutrient Utilization and Amino Acid Digestibility of Dairy Calves Fed Milk Replacers Containing Different Amounts of Protein in the Preruminant Period. Asian-Australas. J. Anim. Sci..

[14] Kertz A.F., Hill T.M., Quigley J.D., Heinrichs A.J., Linn J.G., Drackley J.K. (2017). A 100-Year Review: Calf nutrition and management. J. Dairy Sci..

[15] Chagas J.C.C., Ferreira M.A., Faciola A.P., Machado F.S., Campos M.M., Entjes M.R., Donzele J.L., Marcondes M.I. (2018). Effects of methionine plus cysteine inclusion on performance and body composition of liquid-fed crossbred calves fed a commercial milk replacer and no starter feed. J. Dairy Sci..

[16] Hill T.M., Aldrich J.M., Schlotterbeck R.L., Bateman H.G. (2007). Amino Acids, Fatty Acids, and Fat Sources for Calf Milk Replacers. Prof. Anim. Sci..

[17] da Silva J.T., Manzoni T., Rocha N.B., Santos G., Miqueo E., Slanzon G.S., Bittar C.M.M. (2018). Evaluation of milk replacer supplemented with lysine and methionine in combination with glutamate and glutamine in dairy calves. J. Appl. Anim. Res..

[18] Gelsinger S.L., Heinrichs A.J., Jones C.M. (2016). A meta-analysis of the effects of preweaned calf nutrition and growth on first-lactation performance 1. J. Dairy Sci..

[19] Quigley J.D., Schwab C.G., Hylton W.E. (1985). Development of Rumen Function in Calves: Nature of Protein Reaching the Abomasum. J. Dairy Sci..

[20] Schwab C.G., Broderick G.A. (2017). A 100-Year Review: Protein and amino acid nutrition in dairy cows. J. Dairy Sci..

[21] Kung L., Rode L.M. (1996). Amino acid metabolism in ruminants. Anim. Feed Sci. Technol..

[22] Velle W., Sjaastad V., Aulie A., Grønset D., Feigenwinter K., Framstad T. (1997). Rumen Escape and Apparent Degradation of Amino Acids after Individual Intraruminal Administration to Cows. J. Dairy Sci..

[23] Volden H., Velle W., Harstad O.M., Aulie A., Sjaastad Ø.V. (1998). Apparent Ruminal Degradation and Rumen Escape of Lysine, Methionine, and Threonine Administered Intraruminally in Mixtures to High-Yielding Cows. J. Anim. Sci..

[24] Sancanari J.B.D., Ezequiel J.M.B., Galati R.L., de Vieira P.F., Seixas J.R.C., Santamaria M., Kronka S.N. (2001). Efeito da metionina protegida e não protegida da degradação ruminal sobre a produção e composição do leite de vacas holandesas. Rev. Bras. Zootec..

[25] Lapierre H., Pacheco D., Berthiaume R., Ouellet D.R., Schwab C.G., Dubreuil P., Holtrop G., Lobley G.E. (2006). What is the true supply of amino acids for a dairy cow?. J. Dairy Sci..

[26] Kertz A.F., Prewitt L.R., Everett J.P. (1979). An Early Weaning Calf Program: Summarization and Review. J. Dairy Sci..

[27] Elsohaby I., McClure J.T., Waite L.A., Cameron M., Heider L.C., Keefe G.P. (2019). Using serum and plasma samples to assess failure of transfer of passive immunity in dairy calves. J. Dairy Sci..

[28] Association of Official Analytical Chemists (AOAC) (2012). Official Methods of Analysis.

[29] Larson L.L., Owen F.G., Albright J.L., Appleman R.D., Lamb R.C., Muller L.D. (1977). Guidelines Toward More Uniformity in Measuring and Reporting Calf Experimental Data. J. Dairy Sci..

[30] Chaney A.L., Marbach E.P. (1962). Modified Reagents for Determination of Urea and Ammonia. Clin. Chem..

[31] Quigley J.D. (1996). Influence of Weaning Method on Growth, Intake, and Selected Blood Metabolites in Jersey Calves. J. Dairy Sci..

[32] Ferreira L.S., Bittar C.M.M.M. (2011). Performance and plasma metabolites of dairy calves fed starter containing sodium butyrate, calcium propionate or sodium monensin. Animal.

[33] de Paula M.R., Oltramari C.E., Silva J.T., Gallo M.P.C., Mourão G.B., Bittar C.M.M. (2017). Intensive liquid feeding of dairy calves with a medium crude protein milk replacer: Effects on performance, rumen, and blood parameters. J. Dairy Sci..

[34] Bittar C.M.M., Gallo M.P., Silva J.T., de Paula M.R., Poczynek M., Mourão G.B. (2020). Gradual weaning does not improve performance for calves with low starter intake at the beginning of the weaning process. J. Dairy Sci..

[35] Muller L.D., Rodriguez D. (1975). Methionine Hydroxy Analog Supplementation of Low Protein Calf Rations. J. Dairy Sci..

[36] Hill T.M., Bateman H.G., Aldrich J.M., Quigley J.D., Schlotterbeck R.L. (2013). Evaluation of ad libitum acidified milk replacer programs for dairy calves. J. Dairy Sci..

[37] Von Buenau R., Jaekel L., Schubotz E., Schwarz S., Stroff T., Krueger M. (2005). Escherichia coli strain Nissle 1917: Significant reduction of neonatal calf diarrhea. J. Dairy Sci..

[38] Wenge J., Steinhöfel I., Heinrich C., Coenen M., Bachmann L. (2014). Water and concentrate intake, weight gain and duration of diarrhea in young suckling calves on different diets. Livest. Sci..

[39] Garnsworthy P.C. (2005). Calf and Heifer Rearing: [Principles of Rearing the Modern Dairy Heifer from Calf to Calving].

[40] Khan M.A., Lee H.J., Lee W.S., Kim H.S., Ki K.S., Hur T.Y., Suh G.H., Kang S.J., Choi Y.J. (2007). Structural Growth, Rumen Development, and Metabolic and Immune Responses of Holstein Male Calves Fed Milk Through Step-Down and Conventional Methods. J. Dairy Sci..

[41] Khan M.A., Lee H.J., Lee W.S., Kim H.S., Kim S.B., Ki K.S., Ha J.K., Lee H.G., Choi Y.J. (2007). Pre- and Postweaning Performance of Holstein Female Calves Fed Milk Through Step-Down and Conventional Methods. J. Dairy Sci..

[42] Khan M.A., Lee H.J., Lee W.S., Kim H.S., Kim S.B., Park S.B., Baek K.S., Ha J.K., Choi Y.J. (2008). Starch Source Evaluation in Calf Starter: II. Ruminal Parameters, Rumen Development, Nutrient Digestibilities, and Nitrogen Utilization in Holstein Calves. J. Dairy Sci..

